# The effect of sleeping position on heart rate variability in newborns

**DOI:** 10.1186/s12887-020-02056-2

**Published:** 2020-04-13

**Authors:** Petja Fister, Manca Nolimal, Helena Lenasi, Matjaž Klemenc

**Affiliations:** 1 Division of Paediatrics, Department of Neonatology, University Medical Centre Ljubljana, Ljubljana, Slovenia; 2 University Medical Centre Ljubljana, Ljubljana, Slovenia; 3 Institute of Physiology, Medical Faculty, University of Ljubljana, Ljubljana, Slovenia; 4 Department of Cardiology, General Hospital Nova Gorica, Nova Gorica, Slovenia

**Keywords:** Newborn, Heart rate variability, Sleeping positions, Autonomic nervous system

## Abstract

**Background:**

Lower heart rate variability (HRV) in a newborn might represent a risk factor for unfavourable outcome, a longer recovery after illness, and a sudden infant death. Our aim was to determine whether the newborn’s sleeping position is associated with HRV.

**Methods:**

We performed a prospective clinical study in 46 hospitalized cardiorespiratory stable term newborns. During sleeping, we measured the parameters of HRV in four body positions (supine, supine with tilt, prone, prone with tilt).

**Results:**

The TP (total power spectral density) was significantly higher when lying supine in comparison to prone (*p* = 0,048) and to prone with tilt (*p* = 0,046). The HF (high frequency of power spectral density) in the supine position without tilt tended to be higher compared to prone without tilt (*p* > 0,05). The LF (low frequency power) was significantly higher when lying supine compared to prone, both without tilt (p = 0,018). TP and HF showed a positive correlation with gestational but not postmenstrual age (*p* = 0.044 and *p* = 0.036, respectively).

**Conclusions:**

In term newborns, sleeping position is associated with HRV. Higher TP and HF were found in the supine position, which might reflect better ANS stability. Gestational age positively correlated with TP and HF power, but only in supine position.

**Trial registration:**

ISRCTN11702082, date of registration: March, 13th, 2020; retrospectively registered.

## Background

Autonomic nervous system (ANS) plays an important role in extrinsic regulation of the heart rate (HR) in newborns. The interplay between the sympathetic and the vagal modulation on the sinoatrial (SA) node could be studied by analysing heart rate variability (HRV). In the frequency domain analysis of HRV, the powers of the low (LF) and high frequency (HF) components of the HRV spectrum have been shown to reflect the degree of ANS modulation on the level of SA node [1]. While the HF is suggested to reflect mainly the vagal modulation, the LF is predominantly influenced by the sympathetic modulation. Frequency domain analysis could be performed in a narrow (0,04 to 0,4 Hz) and wider range up to 1 Hz. Because newborns have both, higher heart rate and breathing frequency (BF) than adults, the upper frequency limit of the HF component of power spectral density can be up to 1 Hz. Therefore, wider ranges are usually taken into consideration in newborns. HRV could be regarded as a useful physiological parameter reflecting the responsiveness of the ANS to environmental factors.

Higher HRV might predict a better outcome of illness [2–4]. Massaro et al. conducted a study on 20 newborns with hypoxic ischemic encephalopathy treated with hypothermia and showed that newborns with worse neurological outcome (death in neonatal period or Bayley Developmental Index scores >2SD below the mean at 15-month follow-up) had lower HRV [4]. Lower HRV is suggested to be one of the risk factors for longer recovery after illness [2] and an index of illness severity [2, 5–7]. Griffin et al. showed that a sudden fall of HRV might be used as an early marker of sepsis [6]. Moreover, preterms and newborns with congenital heart anomalies exhibit lower HRV. Butera et al. found reduced HRV parameters in 4–7-year-old children with tetralogy of Fallot who underwent surgery at the age of 2 years in comparison to controls [8]. Moreover, Faye et al. showed that newborns with high levels of pain assessment score postoperatively exhibited lower high frequency variability and therefore, lower HRV in comparison with those with lower values of pain assessment score [9]. Snedec et al. discovered that TP values inversely correlated with the duration of intensive care unit treatment of critically ill newborns and the duration of mechanical ventilation needed [10]. They also suggested that lower HRV parameters during transport could imply their response to stress. HRV analysis might thus be used as a measure to estimate the severity of illness and newborn’s response to stress [2, 10–13].

Lower HRV has also been suggested to be one of the risk factors for sudden infant death syndrome (SIDS) [14–18]. In addition, sleeping position has been reported to be related to SIDS. Moreover, sleeping position also affected cerebral oxygenation in stable preterms: it was higher in the supine than in the prone position [13, 15].

In the literature, only little information regarding potential correlation between the sleeping position and HRV is available. Ariagno et al. studied 16 preterms at 1- and 3-months’ corrected age, respectively and found significantly lower HRV in time domain analysis in prone position during quiet sleep [19].

Accordingly, the aim of our study was to determine the association of sleeping position on HRV analysed by frequency domain analysis. We also studied potential association between blood oxygenation, breathing frequency (BF), mean arterial blood pressure (MAP) and HRV parameters in different sleeping positions and the correlation between gender, gestational and postmenstrual age (PMA) and any parameters of HRV. We hypothesized that the parameters of HRV might be more favourable for outcome in supine position compared to prone, even more so with tilt.

## Methods

### Patients

We conducted a prospective clinical intervention study on 46 cardiovascular and respiratory stable newborns who had no respiratory and/or hemodynamic support. The newborns were hospitalized at the Neonatal Department of the Division of Paediatrics, University Medical Centre Ljubljana, Slovenia, due to diagnostic procedures. Newborns with hypoxic ischemic encephalopathy (HIE), preterms, and newborns with infection, neurological or congenital abnormalities were excluded. The parents gave their informed consent and the study was approved by the National Ethics Committee (0120–458/2016–3 KME 67/09/16). The investigation conforms to the principles outlined in the Declaration of Helsinki.

### Study setting

**Fig. 1:**
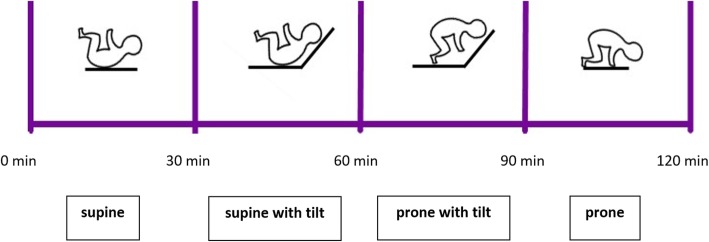
Timeline of the study protocol. After feeding, we started the measurements and the neonate was put in the bed lying supine. Every 30 min the neonate’s position was changed

Before feeding, we installed electrodes to the newborn’s chest. After feeding, we put sleeping newborns in supine position with a 30°head-up tilt of the bed for 30 min. We recorded ECG signal in four positions: the supine without and with tilt and prone with and without tilt (Fig. 1) by using ECG Holter system (Vision 5 L, Burdick, USA). We recorded parameters in every position for at least 30 min, when the newborn was sleeping quietly. Simultaneously, we assessed newborn’s alertness using five stage description [20]. In all positions, the BF was counted (by visualizing the excursions of the thorax) and HR and blood oxygenation measured by pulse oximeter (Intelli Vue MP 50, Philips, Germany) 10 min after changing the lying position of the newborn. Blood pressure (systolic and diastolic) was measured noninvasively using inflatable cuff. Body temperature was measured by infrared non-contact frontal thermometer Veratemp + (Veratemp; USA).

### Data analysis

ECG recordings were analysed by Nevrokard HRV Analysis software (Nevrokard, Izola, Slovenia). The acquired data were checked by a cardiologist and artefacts were excluded. The RR intervals (intervals between two subsequent R-waves of the QRS complex) measured before and after an ectopic beat were replaced by two interpolated RR intervals, which were calculated from a proceeding and a succeeding sinus interval. A fast Fourier transformation (FFT) was used for spectral analysis of HRV. Spectral analysis of HRV was performed in two different frequency ranges, namely from 0,04 to 0,4 Hz (narrow spectrum) and from 0,04 to 1,0 Hz (wide spectrum), the latter because of high BF in newborns. Recording periods of 256 s were analysed, each yielding 512 data points after re-sampling at the 2 Hz frequency. The Hamming windowing function was applied and the Goertz algorithm was used for calculation.

We determined the spectral densities of the narrow spectrum in three different frequency bands: 0,0033–0,04 Hz (very low frequency power - VLF), 0,04–0,15 Hz (low frequency power - LF), and 0,15–0,4 Hz (high frequency power - HF). Likewise, the wider spectrum was divided in three frequency ranges: 0,0033–1,0 Hz (TP - total power), 0,04–0,15 Hz (low frequency power - LF), and 0,15–1,0 Hz (high frequency power - HF) [21]. According to the recommendations of Task Force of European Society of Cardiology and North American Society of Pacing and Electrophysiology [22–24], LF and HF were reported as ratio and in normalized units (LFnu, HFnu), and each component was expressed relatively to the whole HRV spectrum meaning that the VLF component was subtracted LFnu = LF/(total - VLF); HFnu = HF/(total - VLF).

### Statistical analysis

The normality of the sample was tested by Shapiro-Wilk normality test using significance level of 0,05. The descriptive statistics were reported, and the numerical variables summarized as means and standard deviations (SD). The association of the body position with the HRV was verified by the Friedman’s nonparametric test and the post-hoc Wilcoxon tests. For controlling for PMA we have calculated the related samples Friedman’s 2-way analysis of variance by ranks. We used the correction factor by Benjamini and Hochberg for control of false discovery rate in all analyses [25]. The relationship between blood oxygen saturation, BF, MAP, gender, age, and HRV parameters were analysed by Spearman’s correlation coefficient. Adjusted P smaller than 0,05 was considered statistically significant. The statistical analysis was performed by the IBM SPSS Statistics ver. 23.0 software.

## Results

We analysed the data of 46 newborns, 31 (67%) were boys. Because of the missing or inappropriate data, we excluded 5 newborns (1 girl and 4 boys). Demographic data are shown in Table 1 and descriptive statistics of MAP, HR, BF, arterial blood oxygen saturation and temperature are shown in Table 2.

**Table 1:** Perinatal characteristics of the newborns

***N = 46***	Mean ± SD
Gestational age (weeks)	39 ± 1
Postnatal age (days)	11 ± 7
Birth weight (g)	3440 ± 453
Head circumference (cm)	34,9 ± 1,5
Apgar score 10 min	9,5 ± 0,8

**Table 2:** Descriptive statistics of the measured physiological parameters in different sleeping positions

	Supine (S)	Supine with tilt (ST)	Prone with tilt (PT)	Prone (P)	p value^**a**^	p value^**b**^
Arterial blood oxygen saturation (%); Median (IQR)	97,0 (96,0-98,3)	97,0 (96,0-98,0)	96,0 (96,0-97,5)	96,0 (95,0-97,0)	p = 0,001	S vs ST = 0,847 ST vs PT = 0,009* PT vs *P* = 0,037* S vs *P* = 0,004* S vs PT = 0,014* ST vs P = 0,001*
Heart rate (/min) Median (IQR)	136,5 (122,3-145,0)	134,0 (128,0-143,0)	132,5 (124,0-141,3)	130,0 (120,0-138,0)	p = 0,238	NS
Breathing frequency (bpm) Median (IQR)	47 (44–50)	47 (45–50)	45 (43–49)	45 (42,0-47,5)	p = 0,001	S vs ST = 0,039* ST vs PT = 0,001* PT vs P = 0,045* S vs P = 0,001* S vs PT = 0,025* ST vs P = 0,001*
Systolic arterial pressure (mmHg) Median (IQR)	71,0 (62,8-78,3)	73,0 (68,5-82,0)	72,0 (66,0-79,0)	72,0 (67,0-80,0)	p = 0,334	NS
Diastolic arterial pressure (mmHg) Median (IQR)	42,5 (37,5-46,0)	45,0 (40,0-50,0)	41,0 (37,0-46,5)	44,0 (40,0-50,0)	p = 0,637	NS
Mean arterial pressure (mmHg) Median (IQR)	51,0 (47,0-57,0)	54,0 (49,5-59,5)	52,0 (47,3-54,0)	54,0 (48,0-60,0)	p = 0,179	NS
Temperature (°C) Mean (SD)	36,6 ± 0,04	36,6 ± 0,04	36,6 ± 0,04	36,6 ± 0,04		NS

^a^Friedman test

^b^*Willcoxon signed rank test*

*p values < 0,05 (False discovery rate – FDR; corr. Factor Benjamini and Hochberg)

*NS: non- significant*

*Data are presented as median value and IQR (interquartile range), except for the temperature data which are means+/− SD. In the last collon, statistical differences between two positions are presented*

We did not find any significant differences between the individual HRV parameters relative to the spectrum frequency limits; Tables 3 and 4 show the analyses of the wide- and narrow spectrum, respectively. We found significant differences in the TP between supine and prone position (*p* = 0,048) and between supine position and prone position with tilt (p = 0,046). The TP was significantly lower when lying prone in comparison to supine (Table 3).

**Table 3:** HRV parameters in different positions in the wide frequency range 0.04 Hz–1 Hz

	supine (S)	supine with tilt (ST)	prone with tilt (PT)	prone (P)	p value^**a**^	p value^**b**^	p value^**c**^
TP (ms^2^) Median (IQR)	596,5 (395,3-1021,5)	833,7 (251,9-1592,8)	406,0 (249,0-580,0)	327,0 (133,5-715,3)	**p = 0,026**	**S vs ST = 0,387** **ST vs PT = 0,17** **PT vs P = 0,24** S vs PT = 0,046* S vs P = 0,048* ST vs P = 0,036*	**p = 0,018**
HF – (ms^2^) Median (IQR)	113,5 (60,0-214,8)	111,5 (50,0-238,5)	103,0 (47,0-173,0)	93,1 (58,0-237,0)	**p = 0,250**		**p = 0,007**
HF nu – Median (IQR)	30,2 (23,3 – 45,8)	33,8 (22,1 – 53,4)	38,3 (23,5 – 51,3)	49,1 (28,8 – 68,5)	**p = 0,06**		**p = 0,021**
LF – (ms^2^) Median (IQR)	256,0 (150,0-379,8)	298,5 (85,7-428,5)	147,0 (107,4-301,0)	185,5 (60,3-295,8)	**p = 0,015**	**S vs ST = 0,552** **ST vs PT = 0,484** **PT vs P = 0,106** S vs P = 0,019* **S vs PT = 0,261** ST vs P = 0,036*	***p*** **= 0,738**
LF nu – Median (IQR)	68,9 (52,9 – 77,8)	66,9 (56,5 – 78,6)	62,7 (53,0 – 77,9)	50,7 (31,6 – 71,2)	**p = 0,034**	**S vs ST = 0,795** **ST vs PT = 0,75** **PT vs P = 0,123** **S vs P = 0,06** **S vs PT = 0,987** **ST vs P = 0,136**	**p = 0,576**
LF/HF^§^ Median (IQR)	2,4 (1,2-3,4)	1,97 (1,20-3,61)	1,61 (1,03-3,31)	1,04 (0,46-2,48)	**p = 0,039**	**S vs ST = 0,77** **ST vs PT = 0,78** **PT vs P = 0,12** **S vs P = 0,24** **S vs PT = 0,9** **ST vs P = 0,15**	

^a^*Friedman test*

^b^***Willcoxon signed rank test***

^c^***Related-Samples Friedman’s Two-Way Analysis of Variance by Ranks***

*p values < 0,05 (False discovery rate – FDR; corr. Factor Benjamini and Hochberg)

*IQR – interquartile range; HRV – heart rate variability, TP – total power; HF – high frequency power; LF – low frequency;*^*§*^*LF/HF - ratio of the LF and HF*

**Table 4:** HRV parameters in different positions in the narrow frequency range (0,04–0,4 Hz)

	supine (S)	supine with tilt (ST)	prone with tilt (PT)	prone (P)	p value^a^	p value^**b**^
TP (ms^2^) Median (IQR)	498,5 (318–937,5)	581,1 (171,4 – 1105,0)	305,2 (212,0 – 473,0)	253,3 (105,0 – 634,8)	**p = 0,026**	**S vs ST = 0,92** **ST vs PT = 0,42** **PT vs P = 0,29** **S vs P = 0,06** **S vs PT = 0,08** **ST vs P = 0,09**
HF – (ms^2^) Median (IQR)	48,5 (28,8-104,3)	51,0 (19,1 – 116,0)	45,0 (22,0 – 69,4)	46,0 (22,5–77, 8)	**p = 0,081**	
HF nu Median (IQR)	17,35 (12,68 – 26,13)	17,9 (13,8 – 24,4)	20,7 (14,7 – 25,6)	22,5 (16,1 – 36,1)	**p = 0,053**	
LF – (ms^2^) Median (IQR)	256 (150–379,8)	293,0 (85,7 – 293,0)	142,0 (96,0 – 301,0)	174,5 (54,8 – 295,8)	**p = 0,015**	**S vs ST = 0,46** ST vs PT = 0,047* PT vs P = 0,01* S vs P = 0,03* **S vs PT = 0,15** ST vs P = 0,028*
LF nu Median (IQR)	82,7 (64, 6–87, 6)	79,6 (65,1 – 86,2)	78,3 (73,0 – 85,3)	72,2 (55,9 – 83,0)	**p = 0,174**	
LF/HF^§^ Median (IQR)	4,7 (2,3 – 7,1)	4,3 (3,1 – 6,2)	3,6 (2,8 – 5,8)	2,72 (1,8 – 4,9)	**p = 0,039**	**S vs ST = 0,922** **ST vs PT = 1,04** **PT vs P = 0,29** **S vs P = 0,144** **S vs PT = 0,975** **ST vs P = 0,162**

^a^*Friedman test*

^b^***Willcoxon signed rank test***

*p values < 0,05 (False discovery rate – FDR; corr. Factor Benjamini and Hochberg)

*IQR – interquartile range; HRV – heart rate variability, TP – total power; HF – high frequency power; LF – low frequency;*^*§*^*LF/HF - ratio of the LF and HF*

The HF values in the supine position tended to be higher compared to the prone, but not significantly (*p* > 0,05, Table 3). LF was significantly higher in both supine compared to prone (*p* = 0,018, Table 3). LF was also significantly higher in supine with tilt compared to prone (p = 0,036, Table 3). The ratio of the LF and HF tended to be higher in supine in comparison to prone (p = 0,039, Table 3).

No correlations between blood oxygen saturation, BF, MAP and HRV parameters were found. Significantly lower blood oxygen saturation and BF were found when lying prone in comparison with supine (p = 0,002, Table 2).

**Fig. 2:**
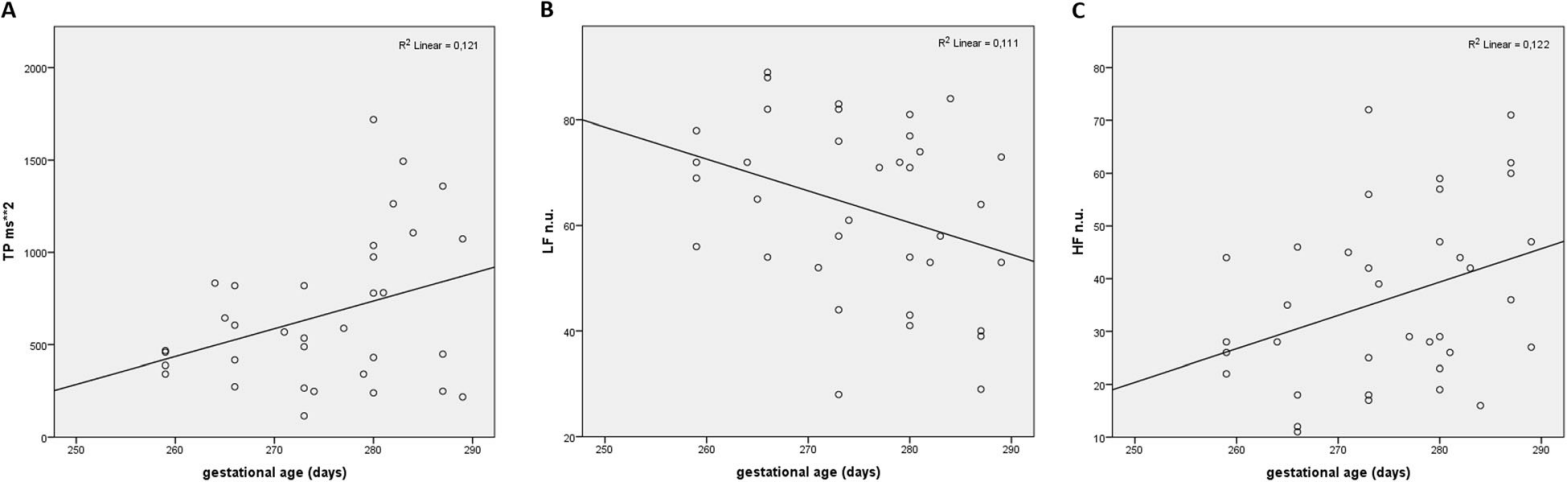
Correlation between: **a** total power (TP) and gestational age, **b** low frequency (normalized units - HFnu) and gestational age and **c** high frequency (normalized units - LFnu) and gestational age in newborns. Pearson’s correlation coefficients and 2-tailed significances: **a:** 0,348 (p = 0,044), **b:** − 0,342 (p = 0,044) and **c:** 0,35 (p = 0,036). Measurements in supine position

We found no correlation between gender and any parameters of HRV. Significant correlations between gestational age and the parameters of HRV were found only in supine (but no other) position: gestational age was positively correlated with TP (R = 0.348, *p* = 0.0449), HFnu (R = 0.350, *p* = 0.036) and negatively with LFnu (R = − 0.342, p = 0.044) (Fig. 2). After considering the PMA of each newborn, none of the HRV parameters correlated to the PMA.

## Discussion

The salient finding of our study is that the term newborn’s sleeping position is associated with HRV as analysed by frequency domain spectral analysis. TP and LF in term newborns are both higher when lying supine in comparison to prone position which might imply an increased responsiveness of the ANS in supine position. To the best of our knowledge, this is the first study that evaluated the effect of sleeping position on HRV analysed by frequency domain spectral analysis in term newborns.

Only little information is available regarding the impact of ANS on the cardiovascular regulation in newborns. Findings show that the activity of the ANS increases with PMA mainly in terms of an increase of reflex vagal activity [26–29]. On the other hand, clinical studies conducted in preterms show greater sympathetic activity, higher HR and less expressed vagal activity as compared to term newborns [27, 28, 30, 31].

In our study, newborns had higher TP HRV when lying supine compared to prone position. Also, the LF was significantly higher in supine in comparison to prone position. Since LF spectrum is supposed to reflect the sympathetic activity [26], we can assume that the cardiac sympathetic modulation is less pronounced in prone position. Similarly, Gabai et al. found reduced HRV parameters analysed in time domain in three-day old term newborns in a prone position [32]. In prone position, they showed a decrease in SDNN (standard deviation of normal R-R) which correlated with TP and also a decrease in short term variability (assessed by pNN50) which correlated with HF. No effect of birth-weight or gestational age on HRV was noted in their study. Similar to our study, Jean-Luis et al. also found both, TP and LF to be significantly higher in supine compared to prone position. Also, similar to our observation, no significant difference was seen in HF [33]. Moreover, Galland et al. also showed lower HRV assessed by the point dispersion of Poincaré plots in prone position in term infants [34]. On the other hand, Ariagno et al. found lower HRV in prone position only in the time domain, but not in the frequency domain: RMSSD (the square root of the mean of the sum of the squares of differences between adjacent R-R), which corresponds to HF was significantly greater in the supine position at both 1 and 3 months’ corrected age, whereas the SDNN was significantly higher in the supine position, but only at 1 month corrected age [19]. These results on increased HF are in agreement with the results of our study and, besides the above speculated sympathetic influence, imply also an important contribution of vagal baroreflex modulation.

Besides assessing the parameters of HRV, we have simultaneously measured the arterial oxygen saturation and BF what was not performed in other available studies. Blood oxygen saturation was significantly lower in prone compared to supine although not clinically important, since in both positions, the measured saturation was above 94%. On the contrary, it has been reported that preterms receiving nasal continuous positive airway pressure (nCPAP) for mild respiratory failure had better arterial blood oxygenation when lying prone [35]. In newborns who were without non-invasive support, we have also observed lower BF when lying prone. Yet, both physiological parameters were within normal limits (BF of the newborn 30–60/min) in both positions and could imply greater impact of the parasympathetic nervous system in prone position [36, 37].

We did not find any correlation between gender and either parameter of the HRV which is in accordance with the findings of Javorka et al. and Yanget al [26, 38].. On the other hand, Nagy et al. found significantly lower HR and lower HRV, expressed as the standard deviation of the HR, in boys [39]. Male newborns had a significantly decreased pNN50 (namely HF) compared to females when lying prone [32].

In our study, newborn’s gestational age but not PMA positively correlated with TP and HF power and negatively with LF power when lying supine but not when lying prone although there is a large variability in the data as shown by the very low R-squared values. Our observations are in accordance with the finding of the Cardoso et al. [40] who have shown higher HRV in older newborns. Gestational age as well as PMA have been shown to be positively correlated with HF and negatively with LF [3, 28, 29, 41]. These findings might implicate that the vagal activity increases with PMA, while the sympathetic modulation of HRV in neonates seems to be less expressed. Chatow et al. confirmed that LF/HF ratio decreases with advanced PMA. Vagal tonus increases with gestational age [42].

Friedman et al. showed that at term, the cardiovascular system is not fully mature yet, and the development continues for several weeks after birth [43]. Bar-Haim et al. found an increase in HF power spectral density also in the period between 4 and 48 months of postnatal age [31]. We might imply that during the period between 37th to 41st weeks the vagal influence becomes more expressed. Interestingly, the correlation between gestational age and the parameters of HRV was significant only in supine position in our study.

Impaired regulation of the cardiovascular system is one of the most important risk factors for SIDS. In infants who later suffered from SIDS, a higher HR and lower HRV were found [44]. Decreased ANS responsiveness has been suggested to contribute to an increased risk for SIDS in infants sleeping in the prone position [16]. In our study, we did not find any significant differences in basal HR in different sleeping positions. On the other hand, we found higher TP and LF in supine position. According to our results we may speculate that sleeping in a supine position could have some advantages in prevention of SIDS.

Besides parameters of HRV, increased arterial blood oxygen saturation in supine additionally speaks in favour of supine over prone position, which is in concordance with some previous studies [12, 15]. Fyfe et al. discovered that cerebral perfusion in preterm infants was significantly lower when lying prone compared with supine in both – active and quiet sleep stages. In accordance with our study, they found lower blood oxygen saturation in newborns when lying prone during a quite sleep at 2 to 4 weeks and at 5 to 6 months of age (*P* < 0,05) [15].

A potential limitation of our study is an intermittent and not a continuous measuring of the BF, MAP, and lack of electroencephalographic data of the sleep stages. Second limitation is a rather small sample size: had we had a larger sample, we could have compared more variables such as the Apgar score, and the type of the childbirth to the variables of HRV. Additional limitation is a rather heterogeneous PMA of the included newborns at the time of the HRV measurement. Yet, as all newborns were older than 5 days, we might assume that they have already overcome the transitional period of hemodynamic adaptations. It might be possible that the sequence in which the positions were applied could be a confounding variable, but to test this hypothesis, too many possible variants should have been tested so we decided for the decribed protocol.

## Conclusion

Our study showed that newborn’s sleeping position is associated with HRV. Higher TP and LF of the HRV analysed in the frequency domain were found in the supine position, reflecting ANS stability. We found a positive correlation between newborn’s gestational age and TP and HF and a negative correlation with the LF in supine, implying an important contribution of the vagal modulation of the HR in supine position. The study might imply that the newborn’s supine position is more favourable in comparison to prone, at least with regard to HRV.

## Data Availability

All data generated or analysed during this study are included in this published article [and its supplementary information files].

## Abbreviations

ANS: autonomic nervous system
HF: high frequency power spectrum
HRV: heart rate variability
LF: low frequency power spectrum
TP: total power
SIDS: sudden infant death syndrome
PMA: postmenstrual age
nu: normalized units
